# Direct construction of sparse suffix arrays with Libsais

**DOI:** 10.1186/s12859-025-06277-z

**Published:** 2025-10-17

**Authors:** Simon Van de Vyver, Tibo Vande Moortele, Peter Dawyndt, Bart Mesuere, Pieter Verschaffelt

**Affiliations:** 1 Department of Mathematics, Computer Science and Statistics, Faculty of Sciences, Ghent University, Ghent, 9000 Belgium; 2 Department of Biomolecular Medicine, Faculty of Medicine and Health Sciences, Ghent University, Ghent, 9052 Belgium; 3 VIB-UGent Center for Medical Biotechnology, VIB, Ghent, 9052 Belgium

**Keywords:** Bioinformatics, Sparse suffix array construction, Libsais, String matching, Text encoding, C programming language, Pattern matching, Bit-packing, Memory-efficient text indexing

## Abstract

**Background:**

Pattern matching is a fundamental challenge in bioinformatics, especially in the fields of genomics, transcriptomics and proteomics. Efficient indexing structures, such as suffix arrays, are critical for searching large datasets. A sparse suffix array (SSA) retains only suffixes at every *k*-th position in the text, where *k* is the sparseness factor. While sparse suffix arrays offer significant memory savings compared to full suffix arrays, they typically still require the construction of a full suffix array prior to a sampling step, resulting in substantial memory overhead during the construction phase.

**Results:**

We present an alternative method to directly construct the sparse suffix array using a simple, yet powerful text encoding. This encoding reduces the input text length by grouping characters, thereby enabling direct SSA construction by extending the widely used Libsais library. This approach bypasses the need to construct a full suffix array, reducing memory usage and construction time by 50 to 75% when building a sparse suffix array with sparseness factor 3 or 4 for various nucleotide and amino acid datasets. Depending on the alphabet size, similar gains can be achieved for sparseness factors up to 8. For higher sparseness factors, comparable performance improvements can be obtained by constructing the SSA using a suitable divisor of the desired sparseness factor, followed by a subsampling step. The method is particularly effective for applications with small alphabets, such as a nucleotide or amino acid alphabet. An open-source implementation of this method is available on GitHub, enabling easy adoption for large-scale bioinformatics applications.

**Conclusions:**

We introduce an efficient method for the construction of sparse suffix arrays for large datasets. Central to this approach is the introduction of a simple text transformation, which then serves as input to Libsais. This method reduces the length of both the input text and the resulting suffix array by a factor of *k*, which improves execution time and memory usage significantly.

**Supplementary Information:**

The online version contains supplementary material available at 10.1186/s12859-025-06277-z.

## Background

Efficiently matching large amounts of short sequences against large reference databases is a fundamental challenge in bioinformatics, particularly in the fields of genomics, transcriptomics, and proteomics. As datasets grow in size and complexity, this challenge becomes increasingly important. Over the years, a lot of specialized algorithms, such as the Knuth–Morris–Pratt [1] (KMP) and Boyer–Moore–Horspool [2] algorithms, have been developed. These algorithms match sequences (consisting of $\:m$ characters) in a text (consisting of $\:n$ characters) in a worst case time complexity of $\:O(n\:+\:m)$ and $\:O\left(n\:m\right),$ respectively. However, such approaches require traversing the entire text to locate all matches, which becomes computationally expensive when the text is large ($\:n\:>>\:m$). To address this, index structures, such as suffix trees [3] and suffix arrays [4], have been introduced. These index structures preprocess the text, allowing short sequences to be matched in $\:O\left(m\right)$ and $\:O\left(m\:log\right(n\left)\right)$ time, respectively.

A trie is a tree-like data structure that stores a set of strings by organizing them according to their shared prefixes. A suffix tree is a compressed trie that stores all suffixes of a given text, allowing patterns to be located by descending the tree from the root along the path matching the pattern. Despite their utility, suffix trees often have a memory footprint that is several times the size of the original dataset, making them less suitable for large bioinformatics datasets. Suffix arrays provide a space-efficient alternative by storing a sorted list of all text suffixes, instead of directly modelling the tree structure. A binary search over the suffix array allows rapid identification of the range of suffixes starting with the desired sequence. Although suffix arrays are more memory-efficient than suffix trees, their memory requirements can still be the limiting factor for large datasets. For example, a suffix array constructed for the UniProt knowledgebase [5] version 2024.04 [6] requires approximately 650 GB of memory, despite the total dataset being only around 82 GB.

Sparse suffix arrays can be used to reduce this memory footprint even further. A sparse suffix array (SSA) retains only suffixes at every *k*-th position in the text, where *k* is the sparseness factor. This means that an SSA of a text of length *n* and sparseness *k* contains only $\left\lceil\:\:\frac{n}{k}\:\right\rceil\:$ entries instead of *n*, reducing space requirements by a factor of *k*. However, lookups in an SSA are slower than in a full SA because multiple shifted queries must be performed to compensate for the reduced sampling, and each candidate match requires additional in-text verification to confirm a full match of the query pattern. The parameter *k* represents a trade-off, balancing reduced memory usage against slower search speed. Algorithm S1 illustrates this search method with pseudocode.

In bioinformatics, there are applications across a range of tasks that require fast and scalable pattern matching on large datasets. Tools like Unipept [7] employ SSAs for peptide-based metaproteomics, enabling rapid and memory-efficient peptide querying against large protein databases. SSAs have also been explored for identifying maximal exact matches (MEMs) [8, 9], a key operation in comparative genomics and seeding-based alignment. MEM finders typically require auxiliary data structures beyond the SSA, such as an Inverse Suffix Array (ISA), which can be constructed with minimal overhead as a post-processing step, or a Longest Common Prefix (LCP) array, which can be built using a dedicated algorithm. Approaches like Sapling [10] have been proposed to accelerate queries on full text indices, such as the SSA, using learned models. Similarly, the developers of Graphite [11] identify the use of sparse suffix arrays as an optimisation, highlighting the continued importance of efficient SSA construction. These diverse applications underscore the utility of SSAs as a lightweight indexing strategy for modern bioinformatics, particularly in contexts where memory constraints pose computational challenges.

Several highly optimized software libraries, such as Libdivsufsort [12] and Libsais [13], are available for the construction of suffix arrays. While very popular, these libraries do not natively support the construction of sparse suffix arrays. The traditional approach to constructing a sparse suffix array involves first constructing the full suffix array and then sampling it. This sampling step selects only every *k*-th entry from the full suffix array to create the sparse suffix array while discarding the rest. Because the full suffix array must still be constructed beforehand, this method does not reduce peak memory usage, which remains as high as that of constructing the full suffix array. To address this limitation, several alternative strategies have been proposed, including Monte Carlo and Las Vegas algorithms [14–16] that construct SSAs probabilistically. These methods offer trade-offs between correctness guarantees and practical performance.

In this article, we introduce an alternative approach for the direct and deterministic construction of sparse suffix arrays. This approach provides fast sparse suffix array construction across various domains, including genomics, transcriptomics, and proteomics. The method reduces memory usage by 63% and execution time by 55% for the SSA construction for the UniProtKB with sparseness factor 3. The method by Ayad et al. [16] represents, to our knowledge, the most recent and relevant approach for direct SSA construction. It is primarily designed for scenarios with high sparseness factors, while our approach is tailored toward lower sparseness factors. Supplementary Figure S2 includes a performance comparison.

## Methods

To optimize memory usage and accelerate the construction of sparse suffix arrays with sparseness factor *k*, we first apply a transformation to the input text. This transformation maps each unique k-mer (k successive characters) to a unique integer, ensuring that lexicographically smaller k-mers correspond to smaller integer values and preserving equivalence. Each non-overlapping k-mer in the input text, taken sequentially from left to right, is then replaced by its corresponding integer according to this mapping. As a result, the effective length of the transformed text is reduced by a factor *k*. One approach to such a transformation is to first assign a unique unsigned integer to each character in the alphabet, using the minimal number of bits necessary for representation. Next, each non-overlapping k-mer is bit-packed [17] into a single unsigned integer, using the smallest possible data size required to represent the k-mer. The bit-packing process is structured such that the leftmost character in each k-mer occupies the most significant bits of the integer, with each subsequent character assigned to the next most significant bits still available. This ensures that lexicographic order is preserved, with smaller k-mers consistently corresponding to smaller integer values. For the canonical amino acid alphabet consisting of 20 different characters, where each character requires 5 bits, grouping $\:k=3$ characters together results in 15 bits per k-mer. These 15 bits can be efficiently stored in a 16-bit unsigned integer within the transformed text. Consequently, the length of the transformed text is reduced by a factor of $\:k=3.$

**Fig. 1:**
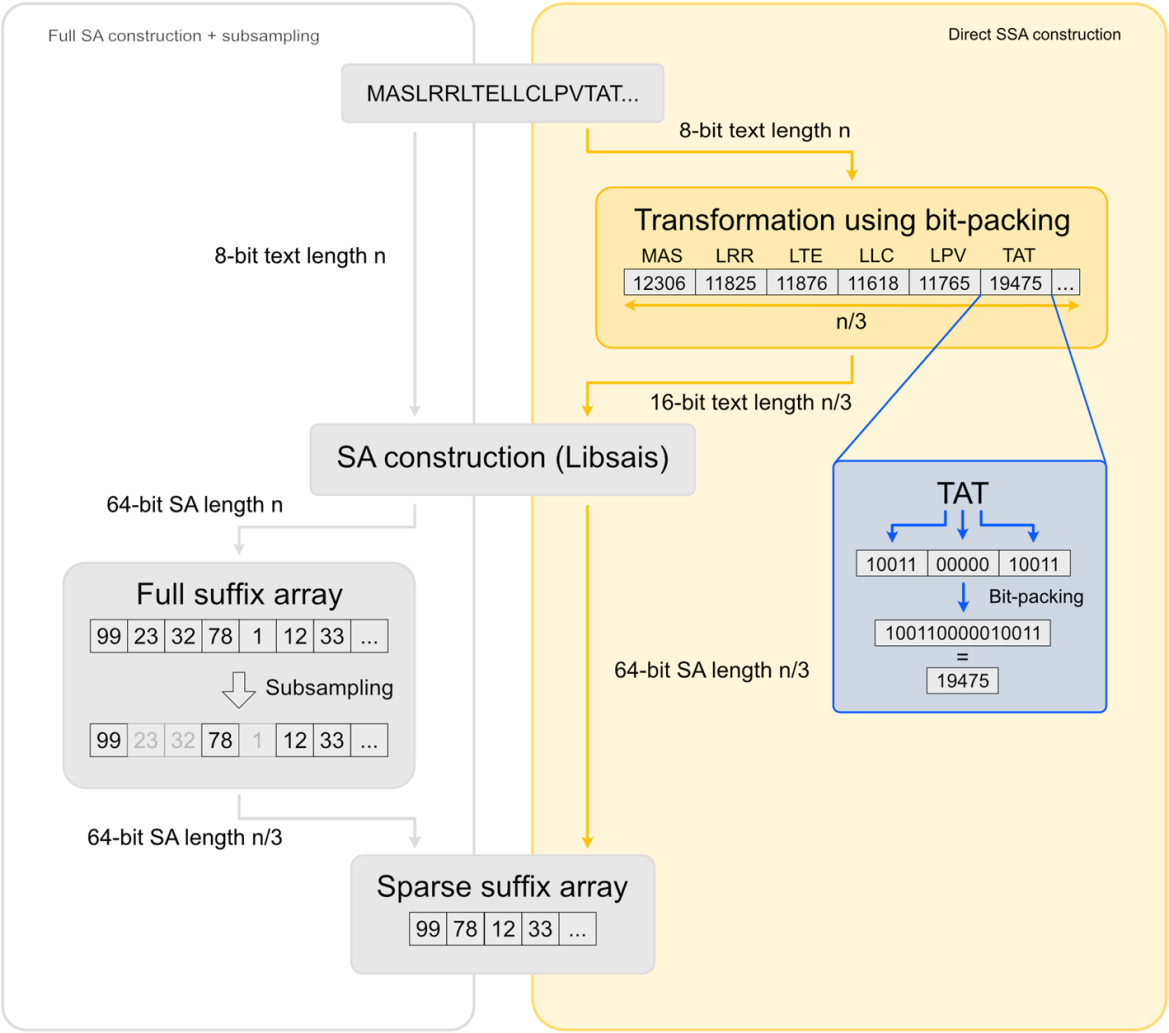
Overview of the standard method (left) using subsampling and the direct method (right) using a text transformation to directly construct the sparse suffix array for sparseness factor 3. The text transformation reduces the length of the text from n to $\:\left\lceil\:\:\frac{n}{3}\:\right\rceil\:$

We then build the suffix array for this sequence of encoded unsigned integers. The result is an SSA of the original input text, equivalent to a sampled SSA derived from a full suffix array. However, it is constructed directly, eliminating the need to first build the full suffix array (see Fig. 1).

This method for direct construction of a sparse suffix array can be used for all suffix array construction libraries, but its efficiency depends on the specific implementation. We use Libsais due to its speed and memory efficiency, and the following complexity analysis is based on its internal algorithm, induced suffix sorting. With Libsais, this approach changes the memory complexity from $\:n+8n+a\sigma\:+b$ to $\:n+8\left\lceil{\frac{n}{k}}\right\rceil+a{\sigma\:}^{k}\:+b,$ where $\:n$ is the length of the original text, $\:a$ and $\:b$ are constants, $\:\sigma\:$ is the size of the alphabet, and $\:k$ is the sparseness factor. The first and second term in each complexity expression correspond to the size of the input text and the SSA, respectively. The third term corresponds to the memory required for bucket allocation performed as part of the induced sorting algorithm, which is the algorithm used internally by Libsais. These buckets store occurrences of each character in the transformed alphabet, where the transformation groups k-mers into a single unit. As a result, the number of distinct characters in the transformed alphabet increases from $\:\sigma\:$ to $\:{\sigma\:}^{k},$ leading to an increase in bucket memory requirements.

For small values of the sparseness factor $\:k$ and small alphabet sizes, this approach yields substantial reductions in both memory usage and execution time, as the effective input length for suffix array construction decreases. However, if $\:k$ or $\:\sigma\:$ is too high, the memory savings may diminish or even increase due to the allocated buckets. By comparison, the traditional method scales more predictably: memory usage grows linearly with the input length and is unaffected by *k*. To handle large *k* with Libsais-packed, it is advantageous to lower the sparseness factor during construction to a divisor of *k* and apply sampling post-construction to achieve the desired sparseness. This flexible approach allows for efficient suffix array construction across a variety of sparseness factors and alphabet sizes.

## Results

We implemented this method in C to ensure efficient performance and seamless integration into existing bioinformatics workflows. Libsais-packed [18], our open-source implementation of the direct construction sparse suffix arrays, demonstrates a large performance boost compared to the traditional method of SSA construction. This approach is particularly effective for datasets with small alphabets, such as genomic, transcriptomic and proteomic sequences.

To evaluate its impact, we present benchmark results for two datasets in the manuscript: UniProtKB [5] 2024_04 [6] and a human reference genome [19]. The UniProtKB database consists of TrEMBL and Swiss-Prot, containing approximately 245 million proteins. All protein sequences are concatenated using a separator character, resulting in a total text length of approximately 82 GB and an alphabet of 25 unique characters. The human reference genome (genome assembly GCF_000001405.40; GRCh38.p14) was downloaded from NCBI and contains a full sequence of approximately 3 GB with 12 unique characters, including the standard nucleobases and ambiguity codes.

Further benchmarks are provided in Supplementary Figure S1 for three additional datasets: *Escherichia coli* (*E. coli*) (GCF_000005845.2; ASM584v2) [20], *Candida albicans* (yeast) (GCF_000182965.3; ASM18296v3) [21], and *Arabidopsis thaliana* (Arabidopsis) (GCF_000001735.4; TAIR10.1) [22]. Supplementary Figure S2 includes a comparative analysis with the method of Ayad et al., evaluated on the human reference genome, the Arabidopsis dataset, and the Swiss-Prot subset of UniProtKB.

The UniProtKB dataset was prepared by concatenating all proteins using a dash (‘-’) as a separation character. All reference genomes were used in their original form without modification. The sparse suffix array construction was evaluated with varying sparseness factors, comparing the standard method using subsampling with the new method using the bit-packing text transformation technique.

## Discussion

**Fig. 2:**
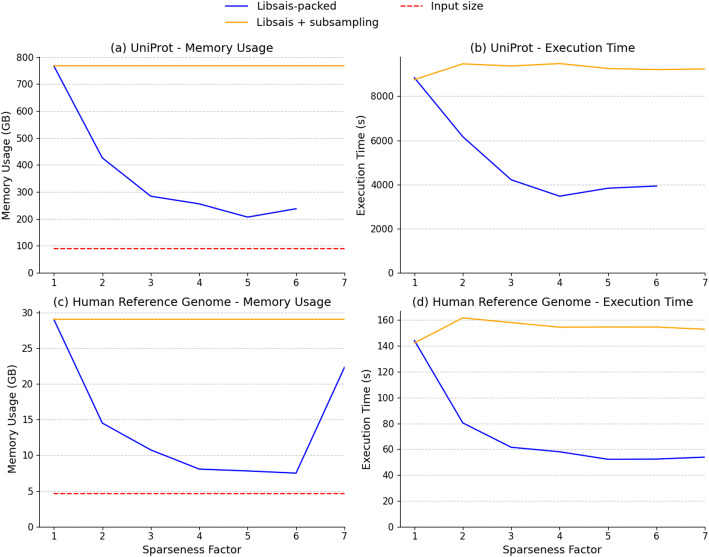
Memory usage (**a**) and execution time (**b**) of the SSA construction for the Uniprot dataset, and memory usage (**c**) and execution time (**d**) of the SSA construction for a human reference genome with varying sparseness factors. The yellow lines show the performance of the traditional SSA construction, using full SA construction with Libsais and subsampling, The blue lines show the performance of Libsais-packed, which constructs the SSA directly through a text transformation. The dotted red line shows the input file size

Figure 2a and b illustrate the execution time (a) and memory usage (b) for sparse suffix array construction on the UniProtKB dataset. Direct construction with Libsais-packed substantially reduces memory usage, with a peak reduction of 73% at sparseness factor 5 compared to the standard approach. As the sparseness factor increases, memory consumption drops sharply at first but starts rising again at sparseness factor 6, reflecting the growing overhead of handling an expanded alphabet in the transformed text. Execution time follows a similar trend, with the method achieving its peak reduction of 60% at sparseness factor 4. Beyond this point, the gains diminish as the alphabet size increases, introducing additional processing overhead. These results demonstrate that increasing the sparseness factor improves efficiency. However, once the alphabet size expands too much, it introduces overhead, reducing the memory and time savings.

Figure 2c and d show the execution time (c) and memory usage (d) for sparse suffix array construction of the human reference genome under the same conditions as for the UniProtKB database. The overall trends mirror those observed for the UniProtKB dataset. Memory usage initially decreases steeply for direct construction, reaching its lowest point at sparseness factor 8, where it is reduced by 86%. Beyond this point, the growing alphabet size leads to increased memory requirements. Similarly, execution time improves as the sparseness factor increases, with a peak reduction of 68% at factor 11, before the overhead from the expanded alphabet begins to counteract the gains, though it remains faster than full suffix array construction followed by subsampling.

Benchmarking across different reference genomes (supplementary Figure S1) shows that the optimal sparseness factor for performance depends primarily on the complexity of the alphabet. For limited alphabets, such as in *E. coli*, higher sparseness factors can be used effectively since alphabet expansion overhead remains small. In contrast, for larger alphabets, such as those in *C. albicans* or *A. thaliana*, the optimal factor is lower to avoid excessive bucket memory usage. A practical starting point for new datasets is to select *k* such that $k\cdot\:\sigma$ is close to 20, and then adjust as needed to balance efficiency and memory use.

## Conclusions

In this article, we introduced an efficient method for the construction of sparse suffix arrays for large datasets. Central to this approach is the introduction of a simple text transformation, which encodes k characters of the input text into a compact representation, which then serves as input to Libsais. This method reduces the length of both the input text and the resulting suffix array by a factor of k. Libsais-packed [18] is a C implementation of this method that can be used as a drop-in replacement for Libsais.

Libsais-packed delivers significant improvements in both memory usage and execution time for the construction of a sparse suffix array, as long as the alphabet size remains manageable, such as in genomics and proteomics. The benefits diminish when the alphabet size becomes excessively large, emphasizing the need to carefully select parameters for the specific dataset.

## Supplementary Information

Below is the link to the electronic supplementary material.

**Supplementary Material 1**. The file Supplementary Material (PDF) contains an algorithm for querying the SSA and the results of two additional experiments. The first includes performance benchmarks on three additional datasets (*E. coli*, *C. albicans*, and *A. thaliana*) to evaluate the method across genomes with varying repetitiveness and size. The second provides a comparative evaluation against the method of Ayad et al. over a wide range of sparseness factors.

## Data Availability

The datasets analysed during the current study are available online. The UniProtKB database 2024.04 is available in the UniProt repository, https://ftp.uniprot.org/pub/databases/uniprot/previous_releases/release-2024_04/knowledgebase/ [6]. The human reference genome (genome assembly GCF_000001405.40; GRCh38.p14) is available in the NCBI repository, https://www.ncbi.nlm.nih.gov/datasets/genome/GCF_000001405.40/ [19]. The *E. coli* genome (genome assembly GCF_000005845.2; ASM584v2) is available in the NCBI repository, https://www.ncbi.nlm.nih.gov/datasets/genome/GCF_000005845.2/ [20]. The yeast genome (genome assembly GCF_000182965.3; ASM18296v3) is available in the NCBI repository, https://www.ncbi.nlm.nih.gov/datasets/genome/GCF_000182965.3/ [21]. The Arabidopsis genome (genome assembly GCF_000001735.4; TAIR10.1) is available in the NCBI repository, https://www.ncbi.nlm.nih.gov/datasets/genome/GCF_000001735.4/ [22].
