# Supplementary material for: Direct construction of sparse suffix arrays with Libsais
Source: BMC Bioinformatics. 2025 Oct 17;26:252. doi: 10.1186/s12859-025-06277-z (PMC12535041; doi:10.1186/s12859-025-06277-z)
Supplement: Supplementary file 1 — Supplementary Material 1. The file Supplementary Material (PDF) contains an algorithm for querying the SSA and the results of two additional experiments. The first includes performance benchmarks on three additional datasets (E. coli, C. albicans, and A. thaliana) to evaluate the method across genomes with varying repetitiveness and size. The second provides a comparative evaluation against the method of Ayad et al. over a wide range of sparseness factors. [file 12859_2025_6277_MOESM1_ESM.docx]

Supplementary Material

def querySSA(t, ssa, p, k):
  matches = []
   
  # Query the pattern and its first k suffixes
  for offset from 0 to k-1:
      if offset >= length(p):
        break
       
      # Define the current suffix of the pattern
      suffix = p[offset : ]

      # Find all positions in SSA where P_suffix matches
      ssa_positions = ssa.query(suffix)

      # Verify prefix of matches in the original text
      for pos in ssa_positions:
        if pos >= offset and t[pos - offset : pos] == p[0 : offset]:
            matches.append(pos - offset)

  return matches

*Algorithm S1: Querying a Sparse Suffix Array. Given a pattern p and a sparse suffix array ssa of a text t with sparseness factor k, the algorithm searches for all occurrences of p in t.*


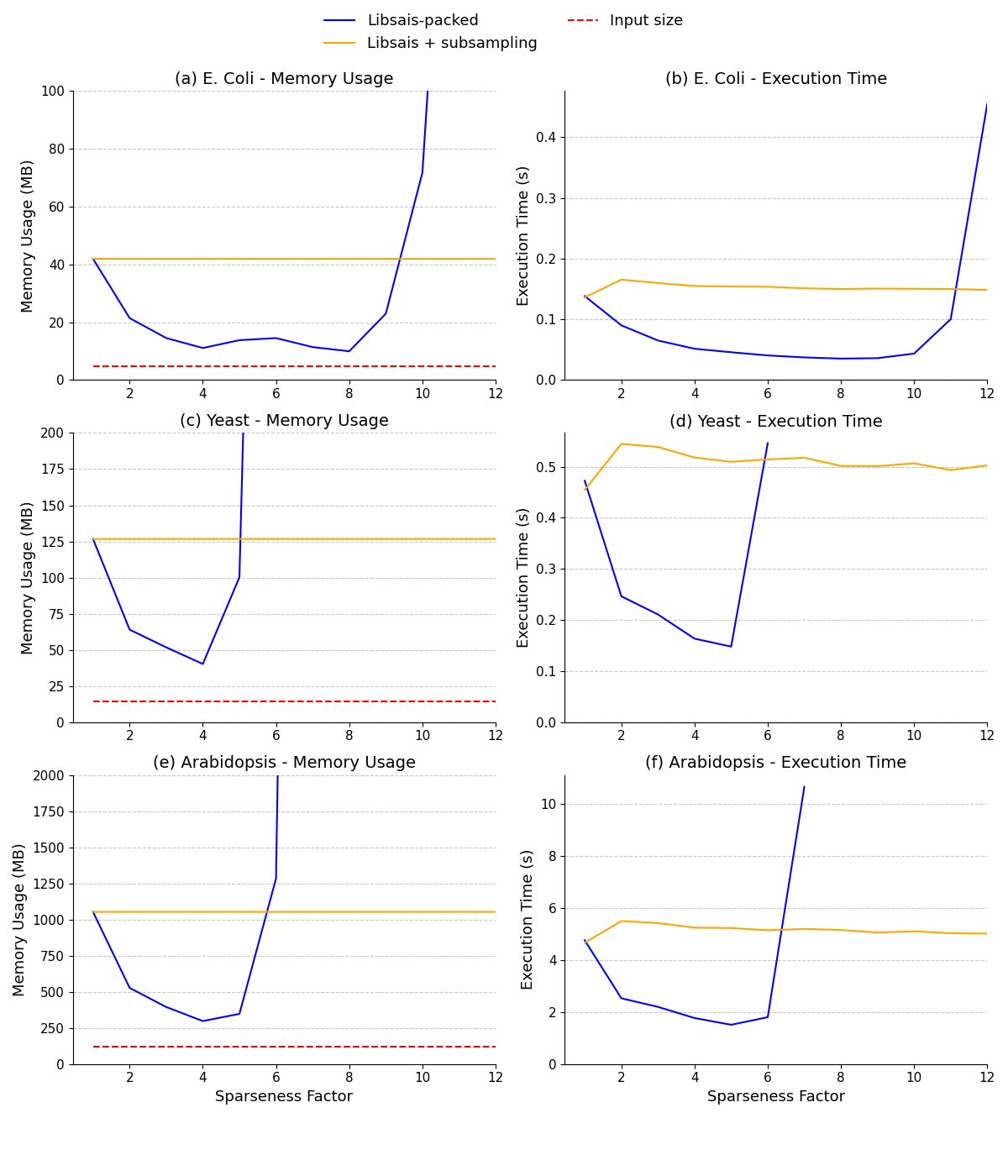


*Figure S1: Memory usage and execution time of the SSA construction for an E. coli genome, a yeast genome and an Arabidopsis genome for varying sparseness factors. The yellow lines show the performance of the traditional SSA construction, using full SA construction with Libsais and subsampling. The blue lines show the performance of Libsais-packed, which constructs the SSA directly through a text transformation. The red dotted lines show the input file size.*

Figure S1 illustrates the memory usage and execution time for sparse suffix array construction on three additional genomes: *E. coli* (S1a and S1b), *C. albicans* (yeast) (S1c and S1d), and *A. thaliana* (S1e and S1f), across varying sparseness factors.

These three datasets were downloaded from NCBI. The *E. coli* genome (assembly GCF_000005845.2; ASM584v2) has a total input size of approximately 4.5 MB with an alphabet consisting of 4 unique characters. The *C. albicans* (yeast) genome (assembly GCF_000182965.3; ASM18296v3) contains approximately 14 MB of sequence data and uses an alphabet of 11 unique characters. The *A. thaliana* genome (assembly GCF_000001735.4; TAIR10.1) consists of roughly 115 MB of sequence data and includes 12 unique characters. These datasets cover a range of genome sizes and complexities, providing a representative benchmark for evaluating the performance of sparse suffix array construction across different biological contexts.

As with the UniProtKB and human genome datasets, Libsais-packed consistently outperforms the traditional method in both metrics. Memory usage initially drops as the sparseness factor increases, reflecting the reduced size of the transformed text. This reduction peaks at different sparseness factors depending on the dataset, after which memory usage rises sharply due to the growing size of the transformed alphabet. Execution time follows a similar trend, with substantial improvements observed for moderate sparseness factors. These results confirm that the benefits of direct SSA construction via text transformation generalize well across diverse genome sizes and alphabet complexities, particularly for datasets with small or moderately sized alphabets.


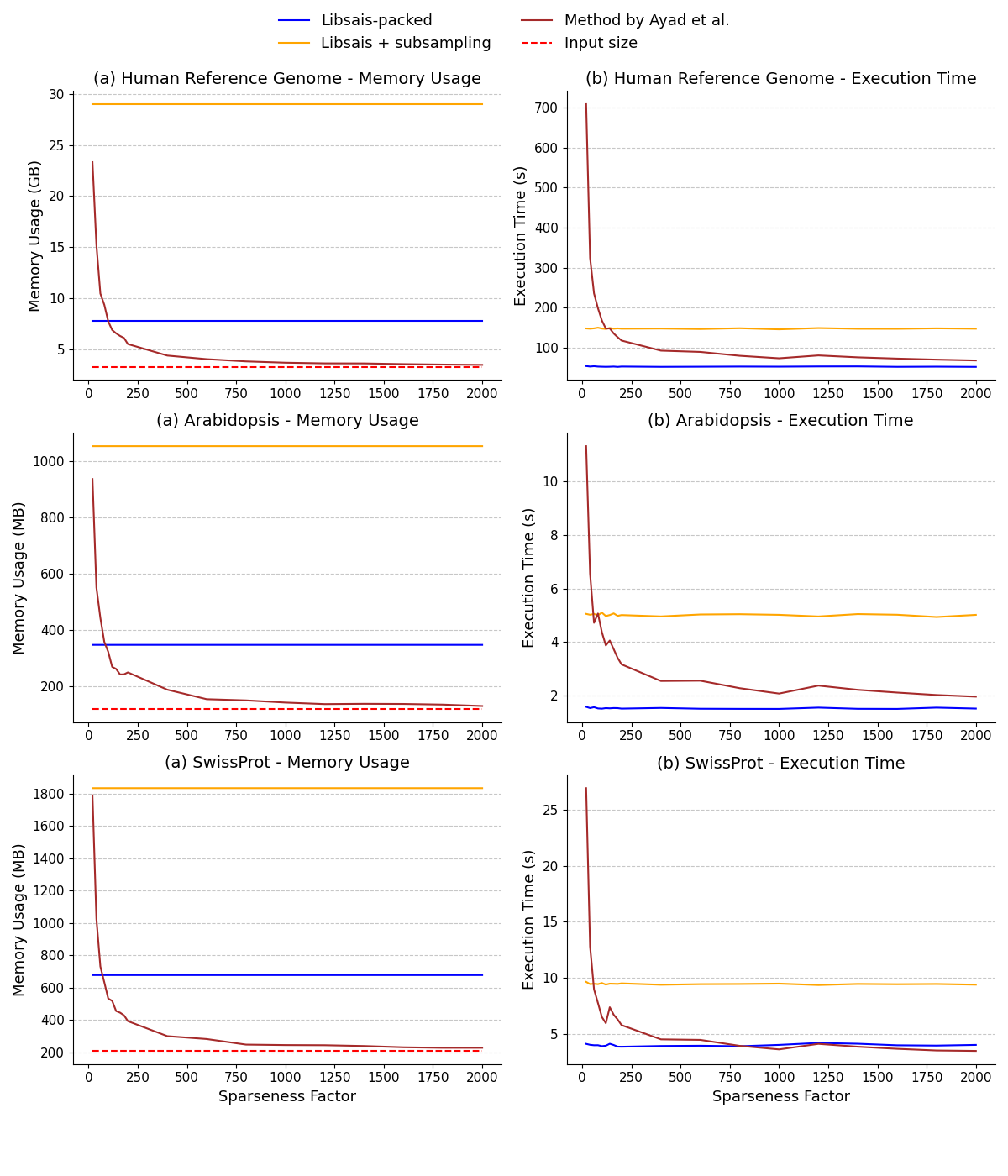


*Figure S2: Memory usage and execution time of the SSA construction for a reference genome, an Arabidopsis genome and Swiss-Prot for sparseness factors ranging from 20 to 2000. The yellow lines show the performance of the traditional SSA construction, using full SA construction with Libsais and subsampling, The blue lines show the performance of Libsais-packed, which dynamically selects a suitable sparseness factor, and performs subsampling afterwards. The brown lines show the performance of the method by Ayad et al. The red dotted lines show the input file size.*

Figure S2 presents a comparative analysis of memory usage and execution time for sparse suffix array construction across sparseness factors ranging from 20 to 2000, using three methods: the approach by Ayad et al., our optimized Libsais-packed implementation, and the unoptimized traditional method, which constructs a full suffix array with Libsais followed by subsampling. In Libsais-packed, the highest divisor of the requested sparseness factor is selected such that the bit-packed k-mer representation does not exceed 20 bits, thereby limiting the overhead introduced by the enlarged alphabet of the transformed text. Subsampling is then applied to achieve the exact requested sparseness factor. Benchmarks were conducted on three datasets: the human reference genome, *A. thaliana*, and the Swiss-Prot subset of UniProtKB. Swiss-Prot is a manually curated subset of UniProtKB, consisting of approximately 199 MB of protein sequence data and an alphabet of 25 unique characters. It is important to note that the method by Ayad et al. also computes the LCP (Longest Common Prefix) array in addition to the sparse suffix array.

Figure S2 shows that the performance trends are consistent across all three datasets, demonstrating the general applicability of the compared methods. Libsais-packed achieves substantial improvements in both memory usage and execution time over the unoptimized baseline, and these gains remain stable as the sparseness factor increases. In contrast, the method by Ayad et al. performs poorly at low sparseness factors, which motivated the choice to begin the benchmarks at a sparseness factor of 20, because differences at lower values would be too pronounced to allow meaningful comparison. As the sparseness factor increases, the performance of Ayad’s method improves rapidly. In terms of memory usage, it surpasses Libsais-packed at around sparseness 100, asymptotically approaching the input size. However, for execution time, it remains slower than the unoptimized method until approximately sparseness 150 and only approaches the performance of Libsais-packed around sparseness 500. Even at the highest tested sparseness factor of 2000, it does not outperform our optimized approach.
